# Cosmetic and Functional Dissatisfaction as Drivers of Elective Hardware Removal After Clavicle Fracture Fixation: A Narrative Review

**DOI:** 10.7759/cureus.107844

**Published:** 2026-04-27

**Authors:** Alexis A Rondinelli, Sarah N Powell, Kenny Thai, Nevin Yazdani, Borna Talle, Nolan Hawkins

**Affiliations:** 1 Office of Research and Innovation, University of New England, Maine, USA; 2 Orthopedics and Rehabilitation, University of Nebraska Medical Center, Omaha, USA; 3 Medicine, University of North Texas Health Fort Worth - Texas College of Osteopathic Medicine, Fort Worth, USA; 4 Medicine, Touro University College of Osteopathic Medicine, Henderson, USA

**Keywords:** clavicle fixation, clavicle implant removal, cosmetic implants, implant removal, plate irritation

## Abstract

Clavicle fractures are a common orthopedic issue requiring a comprehensive approach to management. When surgery is indicated, postoperative concerns and discussions based on psychosocial measures should take place to help patients obtain the best individualized outcomes. Traditional indications for hardware removal include infection, mechanical failure, or significant pain, but patients can elect to undergo removal for non-clinical purposes such as hardware visibility, clothing interference, or activity limitations. These patient-centered indications for removal are currently underreported in large studies and might not be discussed in preoperative and postoperative surgical counseling. This narrative review will synthesize current literature on cosmetic and functional dissatisfaction as drivers of elective hardware removal following clavicle fracture fixation. This review will also evaluate subjective outcomes following implant removal in patients without clinical complications.

## Introduction and background

Clavicle fractures range from simple, nondisplaced fractures to complex, displaced fractures. Clavicle fractures represent the most frequent bone-related injury of the shoulder girdle. The clavicle, or collar bone, is an S-shaped long bone that connects the shoulder blade to the sternum anchoring the arm to the body. It functions as a strut, allowing for free movement of the shoulder and transmission of force from the upper extremity to the axial skeleton. Management strategies depend on the fracture’s classification, displacement, and severity, encompassing both nonoperative and operative approaches. Open reduction and internal fixation (ORIF) is a surgical procedure performed to achieve stable fixation and restore the anatomic alignment of the clavicle, particularly in cases involving displacement, subluxation, or instability. Recent literature has demonstrated an increasing incidence of clavicle fractures accompanied by a corresponding increase in surgical interventions for their management [1,2]. Robinson et al. reported that rates of nonunion are significantly lower after ORIF and further noted that patients expressed greater satisfaction with postoperative outcomes, specifically regarding fewer negative symptoms, such as localized prominence or a residual bump at the injury site [3]. However, one must note that the use of ORIF remains highly variable and subject to regional and institutional preferences.

As surgical treatment becomes more widespread, reports have noted a corresponding rise in postoperative hardware removal. For context, “hardware” refers to the metallic plate and screws used to stabilize the clavicle during ORIF. Because the clavicle lies subcutaneously, this hardware is often palpable and visible depending on the location of the fixation. In a study conducted by Ostergaard et al., routine implant removal was observed in 28% of cases involving clavicle fixation [4]. While hardware removal is clearly indicated in cases of infection, pain, or functional impairment, its role in nonclinical or cosmetic contexts remains poorly defined and widely debated [1]. The existing literature primarily addresses medical necessity and variability in surgeons’ attitudes towards elective removal [5]. Patient-reported outcomes related to hardware removal remain challenging to interpret due to limited sample sizes, case heterogeneity, and the absence of standardized outcome measures that demonstrate cosmetic or quality of life concerns [6]. Current research focuses on two populations: healthy individuals who seek hardware removal for aesthetic dissatisfaction and individuals where cosmetic dissatisfaction is secondary to pain or dysfunction. Both the limited studies focusing on patient-centered outcomes following elective hardware removal and the lack of long-term follow-up data have contributed to our limited understanding of subjective indications for removal. Capturing both surgeons’ perspectives and patient-reported factors surrounding elective hardware removal is critical for establishing evidence-based standardized frameworks that can help better inform shared decision-making in these cases.

## Review

Surgical management of clavicle fractures

The absolute indications for surgical management of clavicle fractures in adults and children are neurovascular compromise, open fractures, scapulothoracic dissociation, and compromised skin integrity [7-9]. Relative indications for surgical management are considered on a case-by-case basis and generally include Neer Type II displaced distal-third fractures, fracture shortening greater than 15 mm, polytrauma, significant neurologic or neuromuscular disorders, and cosmetic issues [9,10]. Given the complexity of determining the appropriateness of surgical intervention, a complete evaluation must be done to ensure that each patient receives the appropriate level of treatment. When surgery is indicated, plate osteosynthesis is considered the primary option for midshaft clavicle fractures [11-14]. However, due to the subcutaneous location of the clavicle, any plating system has the potential for irritation and discomfort. This can be attributed to the various plating technical characteristics, such as plate positioning, dual plating versus single plating, plate thickness, and precontoured plating versus noncontoured reconstruction plating.

Classically, the plate has been positioned on the superior surface of the clavicle. However, the anterior-inferior positioning has been gaining popularity in the past few decades [15-19]. Serrano et al. reported that the odds ratio (OR) for needing implant removal due to plate irritation was five times greater in the superior plate group compared to the anterior-inferior plate group (5 in the anterior-inferior group vs. 25 in the superior group, p < 0.001) [20]. In contrast, Sohn et al. found no significant differences in skin irritation due to plate prominence between superior plating versus anterior-inferior plating (two in the superior group, N=19, vs. one in the anterior-inferior group, N=18, p > 0.999) [21]. Additionally, Jones et al. report that using 2.7-mm dynamic compression plates in the anterior-inferior position resulted in four (3.2%) cases of implant prominence, leading to implant removal in three (2.4%) cases [17]. Plate prominence remains a significant challenge regardless of surgical approach.

Dual plating systems have been increasingly reported on in the surgical management of midshaft clavicle fractures due to the stiffer construct [22]. This technique generally utilizes a reconstruction plate (3.5 mm or 2.7 mm) placed in the anterior-inferior position with a smaller mini-fragment plate (2.4 mm or 2.0 mm) placed superiorly [14]. A systematic review conducted by You et al. found that single plate fixation had a 3.9-fold increase in implant removal rate compared to dual plate fixation with mini-fragment plate or combined fixation with a superiorly placed mini-fragment plate (OR = 3.91, p = 0.003) [23]. Furthermore, Chen et al. reported 0% required implant removal in the dual plating group compared to 8% in the single plating group (p = 0.16) [24]. Czajka et al. also reported a low rate of symptomatic implant removal in patients with dual plating constructs (3.7%) [25]. Dual plating may reduce the incidence of hardware complications by using smaller components compared to traditional fixation.

Regarding plate thickness, a 3.5-mm plate is the most common plate used for clavicle fracture surgical interventions. Galdi et al. found a significantly higher rate of cosmetic acceptance of the 2.7-mm plate cohort compared with the 3.5-mm plate cohort (95% in the 2.7-mm plate cohort vs. 50% in the 3.5-mm plate cohort, p = 0.003) [26]. While there was a decreased rate of implant removal among the 2.7-mm plate cohort, it was not statistically significant (0% in the 2.7-mm plate cohort vs. 17% in the 3.5-mm plate cohort, p = 0.11) [26]. Precontour plating refers to a type of plate that is already in the anatomical shape of the clavicle, eliminating the need for contouring during surgery, unlike a reconstruction plate. Evidence is mixed as to whether precontour plates decrease the incidence of prominent hardware [27,28]. There are a multitude of surgical factors that impact both overall construct stability and eventual bone healing, but they also have longer-term impacts on irritating hardware that may necessitate removal. Technical refinements such as plate thickness and precontour plating are designed to improve anatomical fit and patient comfort. Surgeons must ensure a balance between minimizing the aesthetic profile and maintaining an environment that promotes proper bone healing. With this surgical context established, the subsequent section examines how these hardware-related factors affect the rates of elective hardware removal.

Epidemiology and trends in elective hardware removal

Following clavicle fracture fixation, overall trends in reported elective hardware removal vary. One study found that in a national database containing a large cohort, only 12.7% of 7,826 patients underwent elective hardware removal [29]. This is in contrast with a smaller, single-institution study that showed upwards of 31% of patients undergoing hardware removal postoperatively [5]. Granlund et al. reported that over 90% of removals were for subjective, patient-driven reasons rather than infection or objective mechanical failure [5]. Furthermore, Hulsmans et al. also reported high rates of implant-related irritation that lead to hardware removal, with removal rates of approximately 28% following intramedullary nailing and 35% following plate fixation [1]. These findings accentuate a rising trend between patient-expressed motivators, which typically relate to cosmetics and discomfort, and physician-defined indications, which emphasize hardware removal due to broken implants or medically necessary indications such as hardware-related infections. This dynamic is validated by a prospective review reporting that patient-reported discomfort during daily and physical activities is a common driver of elective hardware removal [30]. More recently, a retrospective study demonstrated the clinical and economic burden of routine asymptomatic hardware removal on both the patients and healthcare system and that there are no universal evidence-based guidelines that govern this practice [31]. Taken collaboratively, these analyses establish that elective hardware removal is not only common after ORIF of clavicle fractures but is also commonly driven by non-clinical reasons [5,30,31]. These findings highlight the need to discuss potential patient-reported dissatisfaction and lifestyle considerations during surgical consultation.

Focusing on demographic factors of patients who request hardware removal following clavicle ORIF reveals significant correlations. A review study containing a cohort (n = 103) with a mean age of 37.8 years (range, 16-75 years) demonstrated a 12.6% hardware removal rate due to discomfort/irritation, with most of the mechanisms of injury being high-energy and/or sports-related [32]. Age has also been correlated with cosmetic satisfaction; younger patients report being more concerned about the cosmetic appearance of their scars and hardware prominence [33]. These psychosocial concerns in the youth population should be discussed when selecting a treatment approach. Gender-based differences in hardware removal are significant, with female patients undergoing hardware removal almost three times more frequently than males (28.6% vs. 11.5%, p < 0.001) [34]. Female patients report higher rates of cosmetic dissatisfaction and mechanical discomfort due to hardware prominence that interferes with certain clothing and accessories [35]. Additionally, Carrillo et al. reported a notable 249% increase in implant removal among adolescent females compared to males, with similar trends reported amongst adult females [29,36,37]. Patients with reduced subcutaneous tissue surrounding the implant site more frequently report dissatisfaction. Ariga et al. reported a significant decrease in rates of implant removal for patients with a body mass index (BMI) of ≥25 kg/m^2^ compared to thinner patients [38]. Of note, this rate was reported for symptomatic hardware removal; however, there is a trend between functionally symptomatic patients and cosmetically dissatisfied patients [38]. This trend likely exists because the source of their complaint is often the prominent hardware itself, yielding both problems [28]. Cosmetic appearance is a notable concern of many patients following operative fixation of clavicle fractures, with Erden et al. reporting a ten percent rate of hardware removal for cosmetic purposes [39]. These numbers are consistent with a meta-analysis reporting a six percent cosmetic dissatisfaction rate [40]. The distribution of patients reporting cosmetic concerns varies significantly based on patient characteristics; however, hardware prominence is the most frequent cosmetic concern in all of these patient groups [41,42]. In many patients, it is likely a combination of cosmetics, pain or irritation, and functional restrictions secondary to implant prominence that is collectively leading to elective implant removal.

Elucidating a single cause can be challenging and likely inaccurate, as patients may have several reasons for requesting a reoperation (Table 1). At any rate, cosmetic satisfaction is an important variable in the treatment plan that should be discussed with patients, including consideration of age, gender, and body habitus as notable factors for cosmetic dissatisfaction and potential hardware removal [33,38]. For patients highly concerned about cosmetic appearance, alterations to the operative approach, including implant choice, can be made to achieve superior cosmesis [27]. These demographic factors and trends in elective implant removal should be considered when planning for surgical intervention of clavicle fractures. Understanding the multifactorial drivers for elective removal is essential for surgeons to weigh the advantages and disadvantages of surgical intervention and anticipate patients' concerns with the ultimate goal of optimal postoperative outcomes and patient satisfaction.

**Table 1:** Overview of reviewed sources related to drivers and outcomes of elective hardware removal after clavicle fixation ADL, activities of daily living; Constant, Constant-Murley Shoulder Outcome Score; DB, database; IM, intramedullary; MSQ, Munich Shoulder Questionnaire; PROM, patient-reported outcome measures; RCT, randomized controlled trial

Study	Design	N (relevant)	Primary Driver(s) for Removal	Key Patient-Reported Outcomes
Hulsmans et al., 2017 [1]	RCT follow-up	116	Implant irritation (66% IM nail, 70% plate)	No long-term functional differences
Granlund et al., 2017 [5]	Retrospective + survey	65 respondents	Pain/soft-tissue irritation; cosmetic concern less common	50% pain resolution; 77% satisfied
Wang et al., 2011 [27]	Retrospective cohort	27 removed	Pain, plate prominence, activity/ADL interference	96% symptom resolution
Naimark et al., 2016 [29]	DB + PROM cohort	7,826 (DB); 73 (PROM)	Mixed; women more likely to request removal	Worse PROMs in removal group
Wurm et al., 2022 [30]	Prospective cohort	27	Plate irritation, activity-related discomfort	Significant improvement in Constant, MSQ
Erden et al., 2021 [39]	Comparative cohort	60	Skin irritation and cosmetic dissatisfaction	Implant removal required in symptomatic cases
Hoogervorst et al., 2020 [40]	Systematic review	1,260	Implant prominence (IM devices)	~6% cosmetic dissatisfaction
Reith et al., 2015 [43]	Survey study	332	Pain, impaired function, patient preference	72–95% symptom improvement
Hambrecht et al., 2024 [44]	Retrospective cohort	82 clavicle	Discomfort (54%), pain (29%)	58.8% satisfied post-removal

Patient-reported outcomes: functional discomfort, lifestyle interference, and satisfaction after removal

Despite the high rate of radiographic and functional success of clavicle fixation, functional discomfort and lifestyle interference remain commonly associated outcomes for many patients. Although these outcomes are subjective and more difficult to assess in a standardized manner, these issues continue to be primary motivators for elective hardware removal.

Wang et al. conducted a retrospective study to compare functional outcomes between plate retention and removal and found that over half of the cohort (56%) underwent hardware removal due to local pain and discomfort, limited range of motion in the shoulder during rigorous sports, or interference with daily activities [27]. Of the 27 patients who had their plates removed, 67% had both discomfort and plate prominence and 33% of patients had problems related to local prominence before removal [27]. After removal, 96% of patients had complete resolution of both issues compared to the group that retained their plates [27]. By comparison, 52% of patients with retained hardware had pain upon impact of the plate, 43% felt that their plates caught on objects, and 19% of patients reported feeling unusual sensations such as a cold patch in the shoulder, aching during cold weather, or numbness from carrying a backpack [27]. Despite the small sample size, it is evident that both functional discomforts, such as seat belts and backpacks, and lifestyle interference, such as sports and work, play a significant role in patient satisfaction due to their effect on the patient's quality of life.

Although functional discomfort and lifestyle interference are primary drivers of elective hardware removal, it remains necessary to evaluate patient-reported outcomes after removal to assess any meaningful benefit of this intervention. Patient-reported benefits of elective hardware removal include a reduction in pain, improved shoulder function, and resolution of cosmetic concerns. Granlund et al. found that of the 36 patients who reported pain, 50% had complete remission of their pain following implant removal [5]. Regarding improved function, Reith et al. found that among patients who underwent hardware removal, 72% reported enhanced function [43]. Additionally, 96% of all responding patients reported that they would opt for implant removal again, underscoring the perceived benefit [43]. Patient satisfaction and qualitative studies play a significant role in determining the real-world impact of elective hardware removal. Granlund et al. reported that 50 (77%) out of 65 patients expressed satisfaction post-removal [5]. Similarly, Hambrecht et al. found that 54% of patients reported a subjective benefit from elective removal [44]. These findings show that satisfaction is not guaranteed and may depend on the specific reason for removal, such as pain versus cosmetic concern, but a significant proportion of patients still report net positive outcomes.

Patients who opt for hardware removal often report relief from symptoms associated with their clavicle implants. Hambrecht et al. reported a patient satisfaction outcome of 58.8% after clavicle implant removal, with symptoms of discomfort (53.7%) and pain (29.3%) being the main reasons for surgery [44]. Additionally, Wurm et al. reported improvements in Constant scores (73.3 ± 14.6 preoperatively vs. 87.4 ± 12.0 postoperatively, p < 0.001), Munich Shoulder Questionnaire (85.0 ± 7.3 preoperatively vs. 91.8 ± 9.0 postoperatively, p = 0.005), mean visual analog scale score for pain (1.4 postoperatively, p = 0.028), and mean pain levels during sleep (p = 0.018) [30]. However, these tools collectively lack specific evaluations related to more subjective discrepancies such as discomfort, irritation, and cosmetic concerns [45,46]. While some patients report improvements after hardware removal, Naimark et al. found contrasting results [29]. In their cohort, patients who underwent hardware removal reported worse long-term functional and satisfaction outcomes than those who retained their hardware [29]. The divergence in the literature underscores the need for individualized decision-making and further comparative research.

A significant problem regarding elective hardware removal after clavicle fracture fixation is the limited and inconsistent data across studies. With only a handful of publications and mixed findings, it is difficult to make a definitive recommendation on whether hardware should routinely be removed. A recent systematic review of patient-reported outcome measure (PROM) trends identified 17 different outcome measures, with Constant scores being the most common in less than half of the studies, highlighting the need for standardized PROM implementation to facilitate appropriate comparisons [47]. Moreover, a multicenter cross-sectional study evaluating 619 patients with lateral clavicle fractures revealed that cosmetics and satisfaction were important primary outcomes and found that 54% of patients required elective removal at long-term follow-up, reinforcing the problem this review addresses [48]. Current evidence suggests that patient preference and subjective symptom burden are central to decision-making. However, better prospective trials, standardized PROM use, and long-term follow-up are needed to clarify the risks, benefits, and ideal timing of removal (Table 2).

**Table 2:** Comparison between cosmetic and functional drivers of elective hardware removal after clavicle fixation ADL, activities of daily living; ROM, range of motion

Driver Category	Specific Patient Concerns	Key Supporting Studies	Typical Outcome After Removal
Cosmetic dissatisfaction	Hardware prominence, visible contour, skin irritation	Erden et al., 2021 [39]; Hoogervorst et al., 2020 [40]	Variable benefit; not universally predictive
Functional discomfort	Pain, irritation, activity limitation, ROM restriction	Wang et al., 2011 [27]; Wurm et al., 2022 [30]; Hambrecht et al., 2024 [44]	Most consistent symptom improvement
Lifestyle interference	Sports limitation, occupational discomfort, ADL issues	Wang et al., 2011 [27]; Wurm et al., 2022 [30]	High patient-perceived benefit
Mixed drivers	Combined cosmetic and functional complaints	Granlund et al., 2017 [5]; Naimark et al., 2016 [29]; Reith et al., 2015 [43]	Outcomes heterogeneous

Perioperative risks, complications, and cost considerations

The anatomy of the clavicle warrants special consideration when planning removal after clavicle ORIF. Preoperative risk factors for refracture following clavicle hardware removal include patient sex and BMI. Female patients showed an approximately 12% higher refracture rate compared to males, as well as a 30% increased risk in females with a BMI of less than 22.73 kg/m^2^ [49]. Additionally, the subclavian vein is particularly vulnerable during surgery on the clavicle, as it runs directly posterior and inferior to the clavicle. Hardware removal carries similar risks as the initial fixation procedure, including hemorrhage and air embolism from vascular injury. Notably, during more medial clavicle fixations, the brachial plexus and subclavian artery lie approximately 12 mm and 17 mm from the clavicle, respectively, and are not protected by the anterior scalene muscle at this level [50]. Preoperative imaging is essential to evaluate for neurovascular alterations caused by the hardware, such as pseudoaneurysm formation.

The cost of hardware removal must also be considered. Elective hardware removal is often covered by insurance, but it still incurs costs to the patient and the healthcare system. Busam et al. estimated the average cost of orthopedic hardware removal to be approximately $5,700, though this varies by location, hospital system, and indication [51]. The decision to remove hardware should be approached with consideration of both clinical and economic factors.

Surgeon perspectives and counseling practices

Surgeons’ perspectives on hardware removal vary across different institutions and geographic regions. Hanson distributed a survey to 730 surgeons, demonstrating wide variation in clinical reasoning for hardware removal, further supporting the lack of consensus regarding indications for hardware removal [6]. The survey found that few surgeons considered removal solely for aesthetic purposes and will primarily perform removal in response to patient-reported symptoms such as pain or discomfort [6]. Therefore, surgeons must consider both clinical and nonclinical motivations when evaluating patient requests for hardware removal.

Proper surgical planning involves discussing the rationale for hardware removal with the patient to ensure their expectations align with the intended outcome. Hambrecht et al. emphasized that having discussions before intervention is crucial for patient-centered care [44]. However, cosmetic dissatisfaction may not be uniformly discussed by surgeons, leading to information that is based on the individual surgeon's opinions and practice rather than standardized evidence-based guidance. The balance between patient autonomy and the ethical obligation to avoid unnecessary surgery presents an ongoing challenge, making the role of shared decision-making increasingly important in elective removal planning.

## Conclusions

Significant gaps persist in the literature on patient-centered outcomes in elective hardware removal following clavicle fracture fixation. Current studies are limited to small cohorts in retrospective studies, leading to a limited ability to extrapolate the data to a wider population base. Another significant literature gap is the lack of standardized PROMs surrounding cosmetic dissatisfaction, functional limitations, and quality of life outcomes following both clavicle fracture fixation and elective hardware removal.

To refine the decision-making process, future research must utilize larger cohorts and prioritize subjective indications for hardware removal. Establishing evidence-based guidelines for these elective procedures will ensure patient expectations are more closely aligned with clinical judgment.
